# Root Vegetables—Composition, Health Effects, and Contaminants

**DOI:** 10.3390/ijerph192315531

**Published:** 2022-11-23

**Authors:** Eliza Knez, Kornelia Kadac-Czapska, Kamila Dmochowska-Ślęzak, Małgorzata Grembecka

**Affiliations:** Department of Bromatology, Medical University of Gdańsk, Gen. J. Hallera Av. 107, 80-416 Gdańsk, Poland

**Keywords:** root vegetables, polyphenols, betalains, carotenoids, dietary fiber, glucosinolates, antioxidant potential, microplastic, toxic metals, nitrates and nitrites

## Abstract

Root vegetables are known all over the world, but they are being less and less consumed by individuals. The main purpose of this article was to summarize the benefits, health effects, and threats associated with the consumption of carrot, celery, parsley, beetroot, radish, turnip, and horseradish. They are characterized by high nutritional value due to their richness in dietary fiber, vitamins, and minerals. One of their most important features is their high content of bioactive compounds, such as polyphenols, phenols, flavonoids, and vitamin C. These compounds are responsible for antioxidant potential. Comparison of their antioxidant effects is difficult due to the lack of standardization among methods used for their assessment. Therefore, there is a need for a reference method that would allow for correct interpretation. Moreover, root vegetables are characterized by several health-promoting effects, including the regulation of metabolic parameters (glucose level, lipid profile, and blood pressure), antioxidant potential, prebiotic function, and anti-cancer properties. However, due to the type of cultivation, root vegetables are vulnerable to contaminants from the soil, such as toxic metals (lead and cadmium), pesticides, pharmaceutical residues, microplastics, and nitrates. Regardless, the low levels of toxic substances present in root vegetables do not pose health risks to the average consumer.

## 1. Introduction

Vegetables are recommended as the basis of a balanced diet. As with all habits, including nutrition and vegetable consumption, children carry them over from home. This has implications for their nutrition in adulthood [1]. Through improper eating patterns, many people suffering from obesity are unaware of the health-promoting effects of vegetables or fruits [2]. Vegetables are good for health and their absence in the daily diet increases the overall cost of health services [3]. Vegetables can be classified based on their botanical origin, hardiness or temperature, and plant part used, i.e., leaves, fruits, or roots. Root vegetables include carrot, radish, potato, yam, ginseng, celery, parsley, and horseradish [4,5,6]. 

Edible roots have some similar nutritional features. All of them constitute a good source of fiber (1.6–7.3 g/100 g). Moreover, they are characterized by a low energy value (16–81 kcal/100 g) due to their high content of water (75.0–95.4%) (Table 1). The caloric value of root vegetables increases in the following order: radish < turnip < carrot < celery < beetroot < parsley < horseradish. One of their most important features is their high content of bioactive compounds, such as polyphenols, phenols, and flavonoids. Moreover, they are rich in vitamin C (3.9 mg/100 g in celery and up to 114.0 mg/100 g in horseradish) (Table 1). Therefore, root vegetables can be considered a biofunctional food. 

Moreover, particular root vegetables are characterized by the presence of compounds specific to them. An example would be carrot, the characteristic feature of which is the high content of β-carotene, a provitamin of vitamin A [7]. Root vegetables are characterized by several health-promoting effects, including the regulation of metabolic parameters (glucose level, lipid profile, and blood pressure), antioxidant potential, prebiotic function, and anti-cancer properties [8,9]. However, it is difficult to compare the results concerning antioxidant potential from different studies due to the various methodologies applied. There is a need to create standardized methods to allow for the comparison of the activity of particular vegetables [10].

Despite their many benefits, root vegetables can also pose a threat, which can be attributed to their ability to accumulate contaminants [11]. Heavy metals, such as cadmium (Cd) and lead (Pb), are continuously detected in root vegetables [12]. Moreover, these vegetables can be also contaminated by pesticides, polycyclic aromatic hydrocarbons (PAHs), and microplastics. All of these are frequently found in roots in various forms and concentrations [13,14,15]. 

Root vegetables, especially beetroot, are characterized by high levels of nitrates and nitrites [16]. These substances have a dual influence on human health. Depending on the dose, they can exert positive or negative effects on health [17,18]. They can be beneficial because they improve blood pressure and increase nitric oxide synthesis [17]. However, too much of them in the diet can contribute to an increased risk of cancer as a result of their conversion into carcinogenic nitrosamines [18]. The concentration of these compounds is determined by the quality of soil and water and use of fertilizers [19]. 

Root vegetables are an often-underestimated group [20]. Therefore, the main purpose of this article was to describe the benefits, health effects, and threats associated with the consumption of these plants. Seven kinds of root vegetables were described, i.e., carrot, celery, parsley, beetroot, radish, turnip, and horseradish, which are conventionally consumed in Europe. They belong to the botanical families *Apiaceae* (carrot, celery, and parsley), *Amaranthaceae* (beetroot), and *Brassicaceae* (radish, turnip, and horseradish) [21,22]. Other root vegetables, including potato, were not taken into account due to their different nutritional value determined by their high content of starch (20.4 g/100 g of product) [6]. In the following sections, the analyzed products are characterized in terms of their biological structure, nutritional value, health effects, and contamination.

## 2. Morphological Structure of Root Vegetables

Holding the plant in the ground and taking up water and mineral salts are the primary functions of roots [23]. The root can also develop into a storage organ and additionally act as a store of spare substances. Such a situation especially occurs in carrots, parsley, beetroots, radishes, celery, turnips, and horseradish, which are the focus of this publication. The ability to accumulate substances is a distinguishing feature of root vegetables. This is of critical importance with regard to the problem of contamination.

Storage roots have strong secondary growth. They are characterized by a small number of xylem elements and a large amount of storage parenchyma to which they owe their shape [24]. Storage roots are most often found in biennial plants. In the first year of growth, these plants produce vegetative organs and store nutrients in their roots. Only in the second year do they produce generative organs, using reserves from the root. The vegetables described here are biennial plants, with vegetables for consumption being harvested from the fields in the first year of the plant’s development. The exception is horseradish, which is a perennial. However, production plantings are carried out as annuals, and only on rare occasions as biennials [24]. 

The described vegetables belong to different botanical families, i.e., *Apiaceae*, *Amaranthaceae*, and *Brassicaceae* (Table 2). The anatomical structure of the storage root can be determined from the outside by a transparent cork, under which the phelloderm is located. In carrots, the phelloderm accumulates spare materials and carotene. Beneath the phelloderm, there is a ring of phloem composed of parenchyma and arranged sieve tubes. The middle part of the root is occupied by the xylem, separated from the phloem by the cambium [23]. 

An abnormal secondary structure of the root is found in beetroot. The growth of the beetroot in thickness is abnormal [23,24]. Unlike other root vegetables, beetroot has several cambial rings. Subsequent rings form outside the first. The second and subsequent cambial rings produce small bundles of phloem and secondary xylem, as well as large amounts of parenchyma filled with spare sugars [23]. 

The different taxonomic orders are not reflected in the way roots grow in thickness. Parsley and carrots belong to the same order and to the same family of *Apiaceae*, but different tissues form the main layers that accumulate reserve substances. In parsley, it is the secondary wood crumb, while in carrots, it is the secondary phloem parenchyma [22]. A large core is, therefore, an undesirable trait in carrots, as studies have shown that it is less nutrient-rich and accumulates more nitrates. As in parsley, the radish cambium produces a thin layer of phloem and a thick layer of secondary wood. The secondary wood contains few vessels and lacks fibers, while by volume, there is a large amount of crumb tissue [25].

## 3. Composition and Antioxidant Potential of Root Vegetables

### 3.1. Carrot

Carrots (*Daucus carota* L.) have been cultivated for more than 1,000 years and are one of the most popular vegetables worldwide. Currently, they are mainly grown in Europe and Asia [20]. In 2020, the greatest producers of carrots and turnips in Europe were the United Kingdom of Great Britain and Northern Ireland (799,715 tons), and Poland (689,100 tons). In Asia, Indonesia (675,760 tons) and Japan (601,016 tons) were ranked as major producers [20]. This vegetable has an average water content of 69.06%–75.3%, depending on the specific variety. The average amounts of protein, carbohydrates, and fiber are as follows: 8.59%, 7.09%, and 7.95% of dry matter, respectively. The fat content is even lower—1.97–4.31% of dry weight (d.w.)—depending on the variety [4,26]. The concentrations of individual components in common carrots are shown in Table 1. 

*Daucus carota* is characterized by a high quantity of antioxidant compounds, such as carotenoids. This is a group of isoprenoids synthesized by all photosynthesizing organisms and those that do not carry out this process (selected bacteria and fungi) [27]. The amount of carotenoids in carrots, including lutein and lycopene and their mutual ratio, determines the color of the ripe vegetable. The greater their content, the more intense the color. The average content of this compound in carrots was determined to be within the range of 5.5–10 mg/100 g of fresh weight (f.w.) [6,28]. Moreover, it was found that storage at a temperature of 20 °C and light can increase the bioavailability of provitamin A—β-carotene in orange carrots [29]. Carotenoids from carrots can increase resistance to oxidative stress. In addition, a natural mixture of these bioactive substances has better antioxidant activity than pure β-carotene [30,31]. The bioavailability of carotenoids from the diet, including carrot, is within the range of 31–50% for non-smoking people and 98% for smokers [32]. This difference may be due to the greater need for antioxidants among smokers. 

Polyphenols are other compounds showing biological activity in carrots. The main compound is hydroxycinnamic acid. It has a content of 3351.5 mg/kg f.w. and represents 93–99% of the total polyphenols [31]. Their amount can be controlled at the cultivation stage, as crop fertilization with nitrogen compounds has been shown to increase the phenol levels in the vegetable [33]. Furthermore, antioxidant compounds, such as polyphenols, in purple Polignano carrots (*Daucus carota L. var. sativus*) were found to increase during storage in a refrigerator and due to factors such as light or temperature. However, the level of ascorbic acid decreases in such situations, but it does not have a negative effect on the overall antioxidant activity [34]. Antioxidant compounds from carrots show high bioavailability. This is due to the low content of antinutrients, such as oxalates, which is noted to be within 5% [35]. Carrot ethanolic extracts showed an antioxidant potential, measured by the hydrogen peroxide scavenging method, of 80.1–86.26% [27].

### 3.2. Celery

Celery (*Apium graveolens* L.) includes three botanical varieties. The most common is rapaceum, or common (root) celery, whose cultivation in Europe in 2019 reached more than 500 tons [36]. Among other root vegetables, celery is characterized by a high sodium content (100 mg/100 g) and relatively high content of carbohydrates (9.2 g/100 g). Moreover, it is a good source of potassium (K) (300 mg/100 g) (Table 1).

Celery is a plant with a high antioxidant potential, which is due to the presence of many compounds, such as phenols, including flavonols (apigenin, kaempferol, and luteolin) and phenolic acids (caffeic acid, ferulic acid, and p-coumaric acid) [37]. The most potent free-radical scavengers are compounds from the flavonol group, which were detected at a level of 4.19 mg/100 g d.w., including luteolin (0.81 mg/100 g d.w.) and apigenin (3.39 mg/100 g d.w.) [38]. The total antioxidant activity of ethanolic extracts of different varieties of this vegetable, measured by the DPPH (2,2-diphenyl,1-picryl-hydrazyl-hydrate) assay, ranged from 79.54 to 105.79 μM Trolox equivalent per 100 g d.w. (TE/100 g d.w.), and the average Trolox equivalent antioxidant capacity (TEAC) value amounted to 92.54 μM TE/100 g d.w. [39]. Pretreatment by washing or soaking shredded celery in water confirmed a reduction in the phenolic content of 30% compared with untreated samples [40]. 

Unfortunately, there are few original papers on common celery and its root. Researchers have focused on the stem and leaves of celery [41]. Future analysis should compare all parts of this vegetable, as they are traditionally consumed.

### 3.3. Parsley

The most common consumer choice of parsley (*Petroselinum crispum* Mill.) is its leaves. However, there are countries, especially in south and east Europe, where the root of parsley is universally used in gastronomy. In Poland, parsley crops are measured in thousands of tons. The root of parsley is usually used to enrich the taste of meals or as a minor component of dishes, e.g., in chicken soup [42]. 

Among all root vegetables, parsley is characterized by a significant content of K (399 mg/100 g) and calcium (Ca) (43 mg/100 g). Moreover, it is also rich in folates (180 µg/100 g). The consumption of 100 g of parsley covers the daily need for folic acid by 30–40% [43]. The composition of parsley root was presented in Table 1. Unfortunately, this root is often overlooked in research in many parts of the world due to its low consumption [20]. For this reason, most studies concern parsley leaves. 

Apigenin is one of the most renowned parsley components because of its health properties, especially its antioxidant potential [44]. The amount found in parsley leaf is 3.69 mmol apigenin-7-O-(2′′-O-apiosyl)glucoside per 100 g of product. In the same study, it was stated that the absorption of this component was nearly 100% because the fraction secreted in urine constituted only 0.22% of the dose administered [45].

Parsley is also characterized by its content of many phenolic acids, such as luteolin, p-coumaroyl, and isorhamnetin [46]. Due to the presence of these compounds, this vegetable exerts a strong antioxidant effect. The degradation of parsley’s bioactive compounds, particularly from the flavonoid group, has been reported during frying or other technological processes with high temperatures. However, a reduction in the amount of cholesterol oxidation products (COPs) in fried omelets was noted when parsley was added [47,48]. 

Parsley leaves can serve as a nutraceutical to enrich other products in antioxidants. The addition of powdered leaves to wheat pasta resulted in a 67% increase in the phenolic content, 146% greater antiradical capacity, and 220% increased reducing power. The antioxidant potential of methanol parsley extracts examined by DPPH, FRAP (free-radical scavenging capacity), and ORAC (oxygen-radical absorbance capacity) amounted to 58.8%, 19.7 mg (TE/100 g d.w.), and 993.2 mg TE/100g d.w., respectively. In a different study, DPPH was determined to be 2.39 μmol TE/100 g d.w. [45,49]. It is difficult to compare these data, as there is no standard method that would allow reliable comparison. Unfortunately, there are no data concerning the antioxidant potential of parsley root.

As with celery, researchers are focused on parsley’s leaves. It is essential to expand knowledge of the root of this vegetable because this part is also commonly consumed in many countries of the world [20].

### 3.4. Beetroot

Beetroot (*Beta vulgaris* L.) is of great interest to consumers in the 21st century due to its pleiotropic health-promoting properties [50]. Among all root vegetables, beetroot is characterized by high quantities of carbohydrates (9.56 g/100 g) and fiber (2.8 g/100 g) [51]. Beetroot is commonly considered a source of iron (Fe), but among root vegetables, it is parsley that contains greater amounts of this microelement [6]. The most abundant element is K (356 mg/100 g in organic beetroots). Moreover, it has been reported that organic beetroots are characterized by higher contents of mineral compounds, such as K, phosphorus (P), Mg, and Ca, than conventional ones [51]. The composition of conventional beetroot is presented in Table 1. 

One of the characteristic features of beetroot is the presence of betalains, i.e., betacyanins (e.g., betanin, betanidin, and isobetanin) and betaxanthins (vulgaxanthin I and dopamine–betaxanthin) [52,53]. Higher concentrations of these substances were found in the peel than in the flesh of the beetroot [51]. Besides their antioxidant potential and anti-inflammatory activity, betalains determine the color of beetroot and can be used as a food pigment [54]. Beetroots contain other bioactive substances, i.e., gallic acid, caffeic acid, p-coumaric acid, and quercetin [8]. When comparing the contents of betalains, phenolic compounds, and antioxidant capacity in conventional and organically grown beetroots, higher concentrations were found in organic beetroots [8]. The presence of compounds from the flavonoid group, saponins and glycosides, was also noted in beetroot. However, the antioxidant properties of beetroot juices, particularly their iron-reducing ability, depend on the betalain content in the product [55]. Additionally, the antioxidant potential increases with higher selenium content [56]. The total phenolic content (TPC) of beetroot methanol extract was to be 313.8 mg gallic acid equivalent per 100 g d.w. (GAE/100 g d.w.). The total content of phenolic compounds, as well as antioxidant potential, can be further increased by storage at 4 °C for 10 days [57]. Another process that affects the content of bioactive substances is fermentation, as the amount of betalains in fermented beetroot is 61–88% lower than that in fresh samples [58].

This root is also a natural source of nitrates and nitrites, which exhibit a dual impact on human health [59]. The average concentration of these substances was found to be 1379 mg/kg, which is the highest amount among all root vegetables [60]. 

Beetroot is quite well known, but the increased interest in pickled beets should be a reason for scientists to enhance knowledge of how this process affects the composition of this vegetable.

### 3.5. Radish

Radish (*Raphanus sativus* L.), among all root vegetables, is characterized by the lowest energy value (16 kcal/100 g), which is because it has the highest content of water (95.3%). Among the analyzed root vegetables, it is characterized by the lowest content of carbohydrates—3.4 g/100 g (Table 1). 

Radish, like the other root vegetables described, has a high antioxidant potential and a significant content of biologically active compounds. The TPC was determined to be 68 mg GAE/100 g f.w. [61]. Flavonoids are the most abundant substances of all antioxidant compounds (38.8%), followed by non-flavonoid polyphenols (8.4%), terpenes and derivatives (8.2%), glucosinolates (GLS) and their breakdown products (5.6%), and hydrocarbons (4.6%) [62]. Glucosinolates are decomposed by myrosinase in Brassicaceae vegetables. This enzyme is activated during plant tissue damage, for example, during cooking or cutting [63]. Isothiocyanates and thiocyanates are breakdown products of GLS with glucoraphasatin, which is found in the highest concentration (163.1 mg/100 g d.w.). Sulforaphane is the main product of GLS breakdown (5.26 mg/100 g d.w.) [62]. The bioavailability of these compounds ranges between 12 and 80% of all GLS present after the consumption of a fresh sample. The large variation between these two values mostly depends on the amount of myrosinase present in the ingested plant and structural properties of each GLS [63]. 

Flavonoids are the major pigment molecules responsible for the color of radish. Pelargonidin-based anthocyanins (callistephin and pelargonin) are mainly found in red radishes, and acylated cyanidin has been identified in purple ones. Moreover, cyanidin o-syringic acid is abundant only in purple radishes [64].

The TPC is a determinant of the antioxidant potential, which is measured for radish by various methodologies. It was noted that the results of the FRAP method were always higher for the leaves than for the roots, but both parts of the plant showed an antioxidant effect. The water, methanol, and acetone extracts were able to reduce ferric ions efficiently and had reducing activities in the range of 1.68–2.83 mM FeSO_4_/g. Similar results were noted in the method with DPPH. The measurements were still solvent-dependent, and the IC50 values (half of the maximum inhibitory concentration) were always higher for the leaves than for the other parts of the plant [65]. 

### 3.6. Turnip

Turnip (*Brassica rapa* L.), like other root vegetables, has a low energy value (28 kcal/100 g). Like radish, turnip is not distinguished by its higher content of any of the macro or micronutrients as compared with other root vegetables. 

Turnip is characterized by a multitude of varieties. The roots can range in color from white to green and to an intense purple. This depends on the concentration of anthocyanins, which are responsible for the intensity of the color [66]. The most common anthocyanins are pelargonidin and cyanidin. In their absence, the root is white [67]. 

As among all *Brassica* vegetables, turnips are characterized by the presence of GLS. The average quantity of GLS in turnips amounted to 2.74 mmol/100 g d.w. [68]. However, sinigrin is not present in these roots [69]. 

Like other root vegetables, turnips are also rich in compounds that increase the plant’s antioxidant potential, such as phenols and flavonoids. The TPC was determined to be 241.27 mg GAE/g, while the total flavonoid content (TFC) was estimated to be 4.45 mg quercetin equivalent per gram (QE/g). Compounds such as catechin (42 µg GAE/g), ferulic acid (265 µg GAE/g), and p-hydroxybenzoic acid (151.25 µg GAE/g) were measured with the highest amounts. Simultaneously, protocatechuic acid (5.60 µg/g d.w.), biochanin A (12.77 µg/g d.w.), and m-coumaric acid (17.95 µg/g d.w.) were measured with the lowest amounts. The oxidative activity of turnips, measured using the DPPH method, was estimated to be 47.5%. The same study showed the importance of the presence and expression of specific genes in the turnip. Transgenic turnips had higher contents of TPC and TFC, and a higher antioxidant potential [67]. 

### 3.7. Horseradish

Horseradish (*Armoracia rusticana* G. Gaertn. et al.) is distinguished by the highest energy value among all root vegetables described (81 kcal/100 g). This is due to its low content of water (75%). The contents of protein (4.5 g/100 g) and carbohydrates (18.1 g/100 g) are also responsible for its high caloric value. A bioactive component that occurs in high concentration is vitamin C (114 mg/100 g). Moreover, horseradish is a good source of K—740 mg/100 g (Table 1).

*Armoracia rusticana*, like other root vegetables of the *Brassicaceae* family, is characterized by a high content of GLS. Their amount in horseradish was determined to be in the range of 0.2–2.9 mmol/100 g d.w. Synigrin was the most abundant compound, accounting for as much as 83% of all GLS [70]. Isothiocyanates formed from the breakdown of GLS are responsible for most of the properties of *Armoracia rusticana*, including its taste and smell. Allyl isothiocyanate is the main compound causing the pungent taste and lacrimation [71]. These are volatile substances; therefore, the storage of horseradish, even in a cool room (5 °C), causes a reduction in their levels, even up to 50% [71,72]. 

Due to the presence of these compounds, horseradish exhibits strong antioxidant properties. The antioxidant potential measured by the DPPH method for the aqueous extract was 48%, while the EC50 value (reducing power) was determined to be 8.6 mg/mL [72].

A summary of the antioxidant potential of all root vegetables is presented in Table 3.

The beneficial qualities of horseradish have been repeatedly confirmed. However, it is not a frequently consumed vegetable due to its specific taste and smell [73]. Future research should focus on minimizing these unfavorable effects while preserving all of its properties. Moreover, as in other vegetables containing GLS, the bioavailability of these substances from fresh samples should be tested, as well as their recovery time in the gastrointestinal tract, interactions with other components of the diet, and influence on gut microbiota.

**Table 3:** Summary of the antioxidant potential of root vegetables.

Vegetable	Folin–Ciocalteu	DPPH	ABTS	Literature
Carrot, root	412.2 mg GAE/100 g d.w.	42.5 µmol TE/100 g d.w.	~6 µmol TE/ g d.w.	[49,74]
Beetroot	313.8 mg GAE/100 g d.w.	568.4 ± 7.6 mg TE/100 g d.w.	35.49 µmol TE/g d.w.	[57,75]
Celery, root	5.2 mg GAE/100g d.w.	105.79 µmol TE/100 g d.w.	1.14 µmol TE/g d.w.	[39]
Parsley, leaf	7.51 mg GAE/100 g d.w.	16.13%	33.85 µmol TE/g d.w.	[76]
Radish, root	341.45 mg GAE/100 g d.w.	1.36 mmol TE/100 g d.w.	n.d.	[77]
Horseradish, root	~1050 mg GAE/100 g d.w.	~7.5 µmol TE/g d.w.	~5.8 µmol TE/g d.w.	[78]
Turnip, root	241.27 mg GAE/100 g d.w.	47.5%	n.d.	[67]

GAE—gallic acid equivalent, TE—Trolox equivalent, d.w.—dry weight, n.d.—no data

## 4. Influence of Root Vegetables on Health

### 4.1. Carrot

Carrots may exhibit prebiotic potential [79]. It was found that, during the digestion of carrot powder in vitro, polyphenols had low recovery up to the large intestine. However, in the colon, fermentation of polyphenols was observed, including conjugated ones. The polyphenols that were released demonstrated antioxidant activity, as well as the ability to inhibit α-galactosidase. At the same time, the composition and diversity of the gut microbiota were regulated by the fermented carrot powder. Such a relationship demonstrated the importance of carrot polyphenols for the colonic microbiota and, thus, for gastrointestinal health. Prebiotic potential was also observed for the fiber associated with polyphenols. In the described study, no fermentation process was carried out in which the fiber could be naturally released from its complexes. Alkaline extraction was used to obtain pure dietary fiber, which promoted the growth of *Lactobacillus rhamnosus* and had the ability to scavenge free radicals [79]. 

Improvement in intestinal function was also demonstrated in rats. Higher stool weight and lower fecal pH were obtained from animals receiving carrot root and pomace preparations. This was observed in the case of both orange and purple carrot preparations. Furthermore, the rats had a better lipid profile and higher antioxidant activity, which was indicated by the decreasing β-galactosidase activity [80].

Similar results were obtained in hamsters, which had lower fecal pH, ammonia levels, and galactosidase activity after consuming carrot and carambola juice [81]. The effect of the insoluble fiber of carrots on the control of the lipid profile was also confirmed in hamsters. Animals receiving pomace from this vegetable showed reduced levels of triglycerides (TG), higher levels of HDL cholesterol, and higher amounts of cholesterol, lipids, and bile acids in feces [81].

Carrots were also studied in terms of their effectiveness in improving female sexual dysfunction. Subjects in the study group received 500 mg of carrot seeds three times a day for 12 weeks, while a control group was treated with a placebo. The results were compared based on completed female sexual function index (FSFI) questionnaires. An improvement in the total questionnaire score of 7.329 ± 0.830 (*p* < 0.001) was observed. No side effects of the described treatment were found, which may be an advantage over pharmacological interventions [82].

Carrots are one of the vegetables rich in carotenoids. The effect of consuming a mix of vegetables and fruits rich in these compounds on their content in the body was studied. It was found that the level of carotenoids increased, but there was no effect on internal lipid oxidation, cholesterol, or triacylglycerols [83]. A strong antioxidant effect of carotenoids was achieved in people by the supplementation of these components [84]. Carrot intake might be inversely associated with cancer, especially prostate, bladder, and breast cancer [7,85,86].

A summary of the health effects of carrot consumption is shown in Table 4.

### 4.2. Celery

Celery affects health mainly in terms of metabolic parameters. Its extracts can exhibit hypotensive effects, as shown both in vitro and in vivo in spontaneously hypertensive rats. In addition, celery extract is able to relax the aortic smooth muscle by blocking the entry of Ca ions into calcium channels [87]. 

A substance extracted from celery, 3-n-butylphthalide (NBP), exhibits potent antioxidant activity. It reduced interleukin 6 (IL-6) and tumor necrosis factor-α (TNF-α), and played an important role in oxidative stress in a rat model of chronic renal failure [88]. 3-n-butylphthalide additionally exhibited diuretic and vasodilatory effects, thus lowering blood pressure [89]. In an animal model of fructose-induced hypertension, celery leaf extract may not have only reduced blood pressure, but also improved cardiovascular parameters and the lipid profile [90].

Celery may reduce the risk of hyperglycemia. Elevated rates of inflammation have been shown to predispose the development of diabetes, and the antioxidant effects of celery compounds may counteract this [91,92]. Additionally, luteolin in celery has been shown in vitro to enhance insulin action through the increased expression of peroxisome proliferator-activated receptor γ (PPARγ) and decreased TNF-α mRNA levels [93]. 

In addition, *Apium graveolens* exhibits bacteriostatic and fungistatic activity in vitro. Its antimicrobial role was noted for all Gram-positive bacteria tested, i.e., *Bacillus aerogenes*, *Bacillus coagulans*, *Bacillus megatarium*, *Bacillus subtilis*, *Lactobacillus lichmani*, and *Staphylococcus aureus*, and the Gram-negative bacteria *Kleibsella pheumoniae*, *Pseudomonas aeruginosa*, *Salmonella typhi*, and *Shigella spp*. A fungistatic effect has been documented for strains such as *Aspergillus niger*, *Aspergillus flavus*, *Candida albicans*, *Cryptococcus neoformans*, and *Trichophyton rubrum* [94]. 

Simultaneously, celery can exert negative health effects. It is a potent allergen that triggers a strong immune system response. The proteins contained in the vegetable (mainly Api g 1 and Api g 5) cause an immediate reaction of the immune system, an IgE-dependent allergy. Its symptoms have been classified as atopic [95,96]. The allergenicity of celery was confirmed by a double-blind, placebo-controlled food challenge. A positive response was observed in 22 of 32 patients with a history of adverse reactions to celery root. Allergic symptoms were varied, where atopy predominated [95]. A case of anaphylaxis after celery consumption was also reported, but this was probably related to cross-allergies to birch and grass pollen. Furthermore, celery allergy is frequently caused by cross-reactivity with the major birch pollen allergen Bet v 1 (*Betula verrucosa*) [97].

Due to the allergenicity of celery, researchers should focus on possible methods to minimize this effect. Fermentation may be a good method of reducing allergenic proteins, as their denaturation might occur in the process, which was discovered in legumes [98]. 

### 4.3. Parsley

Unfortunately, there is a lack of research examining the relationship between parsley root and health. Studies have focused on parsley leaves and concerned cell lines or animals (Table 4). Therefore, future research should focus on roots and compare their effects with those exerted by leaves, described below. 

Parsley leaves exhibit anti-fatigue effects. There were noted improvements in serum fatigue indices among swimming mice that were administered ethanol extracts from this plant. In addition, the same study described the effect of fatigue and the administered extracts on the mice’s microbiota. Among the control group, there was a reduction in the *Firmicutes* to *Bacteroidetes* (F/B) ratio and a decrease in the Shannon index, indicating reduced microbial diversity. Despite fatigue, there were no negative changes in the microbiota in the group of mice receiving compounds isolated from parsley. In addition, *Bifidobacterium* and *Lactobacillus* were detected in higher abundance in the group not subjected to fatigue [99]. Parsley leaves have also exhibited antimicrobial properties in vitro. It was reported that aqueous–methanol extracts of parsley leaves had strong antimicrobial activity. This type of functionality has been noted for microorganisms such as *Listeria monocytogenes, Bacillus cereus*, and *Escherichia coli* [100].

Parsley leaf juice may also exhibit protective effects during toxic metal poisoning. Mice that were exposed to Cd (5 g/kg b.w./day) after receiving parsley juice showed improvement in the negative changes and symptoms caused by poisoning. A reduction in lipid peroxidation, an upregulation of the glutathione concentration in the brain, and positive behavioral changes were observed [101]. 

Crude parsley extract (CAE) was also characterized by antiatherogenic effects. The oral administration of CAE (3 g/kg) to experimental animals inhibited platelet aggregation and prolonged the bleeding time. Such results may be related to the high antioxidant potential of parsley, which affects the normalization of thrombocyte hyperactivation [102].

Parsley is also known for its diuretic effect. The consumption of this vegetable may be useful in prevention and intervention for people with kidney stones [103]. Moreover, the intake of 20 g parsley/10 KJ total energy/day decreases apigenin excretion and increases the activity of certain antioxidant enzymes, such as superoxide dismutase (SOD) [104].

A summary of the influence of root vegetables from the botanical family *Apiaceae* on health is presented in Table 4.

**Table 4:** Influence of root vegetables from the botanical family *Apiaceae* on health.

Vegetable	Type of Study	Form of Vegetable/Compound Tested	Effect	Source
Carrot	In vitro	Carrot powder	Fermentation of polyphenols Antioxidant activity and ability to inhibit α-galactosidase shown by polyphenols Regulation of gut microbiota diversity	[79]
Carrot	In vivo/rats	Carrot root and pomace preparations	Higher stool weight Lipid profile improvement Lower fecal pH Reduction in β-galactosidase activity	[80]
Carrot	In vivo/rats	Fermented carrot juice	Regulation of glucose level Regulation of insulin sensitivity Increased short-chain fatty acids in the small intestine Increased microbiota abundance	[105]
Carrot	In vivo/rats	Carrot seed extract	Increased level of thyroxine Decreased spatial memory and passive avoidance memory	[106]
Carrot	In vivo/hamster	Carrot and carambola juice	Lower fecal pH Reduced β-galactosidase activity	[81]
Carrot	In vivo/women	Carrot seed	Female sexual function index (FSFI) questionnaire improved (mainly higher desire, higher satisfaction, and lower pain involving sexual intercourse)	[82]
Carrot	In vivo/people/ meta-analysis	Various carrot products	Decreased risk of prostate cancer associated with intake of carrots Carrot intake might be inversely associated with prostate cancer risk	[7]
Carrot	In vivo/people/meta-analysis	Various carrot products	Dietary carrot intake associated with decreased risk of breast cancer Studies conducted using various methodologies	[86]
Parsley	In vivo/mice	Polyphenolic fraction of parsley	Decreased depressive behavior (decreased immobility time) Decreased anxiolytic behavior (tendency for discovery in the center and illuminated areas)	[107]
Parsley	In vivo/rats	Polyphenolic fraction of parsley	Antithrombotic effects Reduced venous thrombus formation by 98.2%	[108]
Parsley	In vivo/rats	Parsley leaf extract	Morphology improvement in pregnant diabetic rats Fetuses’ metabolic changes caused by mothers’ diabetes decreased	[109]
Parsley	In vivo/rats	Parsley seed extract	Total cholesterol, triglycerides, LDL cholesterol, and vLDL cholesterol decreased Cholesterol HDL increased	[110]
Parsley	In vivo/rats	Parsley leaf	Parsley did not cause any significant reduction in uric acid levels in serum of normal rats, but significantly reduced uric acid levels in serum of hyperuricemic rats in time-dependent manner	[111]
Celery	In vitro/LNCaP cells	Celery extract	Apoptosis induction Anticancer activity on the human prostatic carcinoma cell line LNCaP Time- and dose-dependent inhibition of cell viability by the extract	[112]
Celery	In vivo/mice	Fermented celery juice	Increased relative abundance of beneficial bacteria in gut microbiota Increased ratio of Firmicutes/Bacteroidetes Decreased relative abundance of harmful bacteria (Alloprevotella and Helicobacter)	[113]
Celery	In vivo/mice	Celery extract	Improved both spatial and non-spatial memories Reduced lipid peroxidation of brain Increased glutathione peroxidase activity	[114]
Celery	In vivo/rats	Celery extract	Decreased infarct volume Improved neuronal density in cortex and hippocampus	[115]
Celery	In vivo/women	Celery seed	Female sexual function index (FSFI) questionnaire improved (mainly higher desire, higher arousal, and lower pain involving sexual intercourse)	[116]
Celery	In vivo/people	Celery leaf extract	Decrease in pre-prandial plasma glucose levels among patients with diabetes No significant increase in plasma insulin levels	[117]
Celery	In vivo/people	Celery seed extract	Systolic blood pressure decreased by 11 mmHg on average Diastolic pressure decreased by 8 mmHg on average	[118]
Celery	In vivo/people	Celery, root	Six of eleven patients with history of allergic reactions to celery showed allergic response to cooked celery Celery remained allergenic even after extended thermal treatment (76.07 min/100 °C) Celery spice was allergenic for patients with allergy to raw celery	[119]

### 4.4. Beetroot

Due to the presence of nitrates, beetroot and supplements made from this vegetable are recognized for their support in improving the respiratory and cardiovascular systems [51,120]. However, the consumption of beetroot has better health properties than supplementation with nitrates [51]. Beetroot juice may support the treatment of asthma and psychological stress by protecting against respiratory infections [121]. It was found that beetroot juice rich in nitrates did not significantly improve the systolic and diastolic blood pressure, heart rate, or the six-minute walk test [18]. However, this preparation increased the score of the Borg Rating of Perceived Exertion (RPE) scale, reflecting higher levels of exercise and physical activity among a group of patients with chronic obstructive pulmonary disease [17]. 

In addition, a systematic review of beetroot juice supplementation in a group of endurance athletes confirmed the hypothesis that the supply of this product can alleviate the effects of hypoxia caused by cardiopulmonary failure [122]. For athletes, beetroot juice supplementation may be important in increasing muscle power in both the concentric and eccentric phases of movement [123]. However, no positive effect of beetroot juice consumption on the physiological, perceptual, or performance responses during submaximal or maximal running exercises on a treadmill or time trial on a bicycle was observed [124,125]. 

However, in a group of healthy elderly subjects (67 years), the consumption of 150 g of beetroot and a medium-sized banana every day for eight weeks reduced their resting blood pressure. The intervention also had an impact on the intestinal microbiota, where a lower relative abundance of bacteria of the genus *Bacteroidetes* and a higher relative abundance of microorganisms of the genus *Alistipes*, as well as an increased Shannon diversity index, were observed [126]. 

Beetroot was also studied repeatedly in terms of its effect on the lipid profile, but the results were contradictory. Differences in the results might be due to the small sizes of the investigated groups (8–64 participants) [127,128,129,130]. In 2022, it was concluded that beetroot consumption is not important in this aspect [131].

Future research should be conducted on larger groups that would allow for the extrapolation of the results to the whole population. 

The summary of beetroot’s impact on health is presented in Table 5.

### 4.5. Radish

The most popular health-promoting use of radish is its impact on glucose levels in diabetic patients. Radish sprouts, due to the presence of isothiocyanates, can reduce the activity of α-amylase and α-glucosidase. As a result, a reduction in glucose absorption and lower blood glucose levels in *Drosophila melanogaster* were noted [132]. Similar changes were observed in rats fed with radish sprouts at 0, 2.5, and 5% of the total diet. Animals in the test group not only had lower blood glucose levels, but also lower insulin and total cholesterol levels [133]. Another study analyzed the hypoglycemic effect of radish root juice in rats. The significance of the product was confirmed and 300 mg of juice per kg of body weight was determined to be the best dose. A 33.4% reduction in glucose levels was observed for such a supply [134]. In addition, antioxidant compounds in radish have beneficial effects on diabetic conditions. These substances, including sulforaphane, induce antioxidant enzymes, such as glutathione transferase [135]. 

The health-promoting effects of this vegetable were also demonstrated in other conditions. It was found that patients who were administered radish juice at 25 mL four times a day for 20 days, along with medication, showed better improvement in jaundice compared with those receiving medication alone [136].

The health properties of radish are mainly determined by its GLS content. Although there are many studies on the assimilation and metabolism of GLS, there is still a need to expand knowledge of their behavior in vivo, as well as their interactions with other food components and influence on gut microbiota. 

### 4.6. Turnip

The consumption of turnips can have cardiorespiratory benefits. Their consumption in a powdered form by healthy individuals for seven days affects hypoxia tolerance. Interestingly, improvements were noted only among women, but not men [137]. It would be interesting to research this aspect with a larger group of study subjects.

Another function of turnips and their components is their cytotoxicity against cancer cells. This is related to the presence of GLS. The IC50 values for the tested varieties are directly proportional to the content of these compounds in the plant [138].

### 4.7. Horseradish

Horseradish has been used since ancient times for its flavorful qualities. In addition, its health-promoting properties were noticed thousands of years ago, and it has been used as a medicinal plant [139]. Its components, GLS and thiocyanates, show pleiotropic effects on health, the most important of which is the antioxidant activity and anticancer function [140]. This was investigated in rats in the context of bladder cancer [63,140]. Allyl isothiocyanate (AITC), found abundantly in horseradish, has a stimulating effect on the activity of phase II detoxification enzymes, such as quinone reductase (QR) and glutathione S-transferase (GST). High doses of AITC (100–200 µg/kg b.w./day) were found to increase QR and GST activity in many organs, while at low doses (5–50 µg/kg b.w./day), the change was observed only in bladder tissues [140]. 

In addition, *Armoracia rusticana* extracts have shown inhibitory activity against lipopolysaccharide (LPS)-stimulated inflammation, as demonstrated on peripheral blood mononuclear cells (PBMCs). Horseradish extract affects the mitogen-activated protein kinase (MAPK) signaling pathway, which is one of the most important cascades causing inflammation. Almost complete inhibition of the MAPK pathway was evident after exposure to 333 µg/mL of this extract [141]. Horseradish has also been used to improve the lipid profile in mice. It was found that animals receiving a diet enriched with this vegetable (10 g/kg of food) showed reduced levels of total cholesterol and triacyloglycerols. Moreover, an increased concentration of bile acids was noted. This is related to the fact that the degradation of cholesterol to bile acids is the main pathway for the elimination of this compound from the body [142].

Due to their properties, GLS and compounds such as AITC could become valuable additives for food enrichment. If a method to neutralize the specific taste and smell of this root was found, it could increase the consumption of horseradish [73].

A summary of root vegetables from the botanical order *Brassicaceae* and their influence on health is presented in Table 6.

## 5. Contaminants

The biological value of root vegetables consumed may be lower due to the content of contaminants, i.e., substances that can be harmful to human health. The accumulation of particular contaminants in root vegetables depends on many factors, such as the pH, ionic strength, soil texture and quality, organic matter content, and time [148]. Heavy metals, nitrates and nitrites, organic compounds, pesticide residues, and mycotoxins may be present in these vegetables [149]. Root vegetables constitute a group particularly vulnerable to the presence of contaminants, as soil can be polluted through sewage sludge or fertilizers [148]. A common soil contaminant is also microplastics, which can migrate inside plants [150]. 

Among the most dangerous contaminants to human health are organic pollutants, such as pharmaceutical residues or polychlorinated biphenyls (PCBs), and other industrial and urban waste materials, such as coal ash, etc., which enter the soil with sewage [151]. Toxic metals, such as Pb and mercury (Hg), take the same pathway as PCBs to enter soils [148]. 

### 5.1. Nitrate and Nitrite

The contents of nitrates and nitrites depend on the soil properties, light conditions, moisture content, growing season, planting density, geographic region, fertilization, harvest date, species, or variety of vegetable [152,153]. These compounds are characteristic of root vegetables, especially beetroot. The adequate daily intake (ADI) of nitrates has been set at 3.7 mg/kg body weight/day, whereas, for nitrites, it has been established at 0.06 mg/kg body weight/day [154]. Nitrates and nitrites are useful as food additives. The maximum permitted level (MPL) of these substances was set at a range of 10–500 mg/kg for a product [155]. What is important is that it has been shown that the consumption of beetroot, unlike supplements, does not pose a risk of exceeding the ADI established for these compounds [51].

The concentration of nitrates also varies in different parts of the plant [156]. Carrots, especially early varieties, tend to accumulate these compounds. Most nitrates are stored in the head and apex of the root, more in the axial cylinder than in the primary cortex. The content of both compounds is higher in the superficial layers of some root vegetables, e.g., beetroot, than in the central parts. Such a relationship does not exist in carrots, in which higher amounts of nitrates and nitrites are found in the core of the root [60]. Moreover, during the cooking process, the level of nitrates decreases significantly [157].

The presence of nitrates in vegetables is particularly related to the type of irrigation. Radish, basil, and cilantro grown in soil irrigated with river water were found to contain these compounds at a level of 103.2 mg/kg d.w., while the level of nitrates in the water was 56.2 mg/L [158]. Despite the high nitrate content, the average consumer is not at risk of exceeding the ADI by consuming even a mixture of all three vegetables tested [158]. The mean content of these compounds in carrots in 64 countries was determined to be 159.41 mg/kg f.w. In addition, random sampling of soil in the city of Sanandaj (Iran) showed that the levels of these compounds ranged from 4.35 to 9.7 mg/kg [159]. 

An adequate nitrogen (mg N/kg) content in vegetables can promote their growth. Nitrates at levels of 150–300 mg/kg d.w. in parsley have been shown to provide optimal growth for its root [9]. In addition, technological processes, such as incubation, cooking, and storage, increase the content of nitrates in root vegetables compared with fresh samples [9]. Beetroot is a rich source of nitrates and nitrites; thus, a powdered form of this vegetable can be used for the preservation of food, for example, sausages [157]. 

Nitrates and nitrites can transform in the body into carcinogenic nitrosamines [18]. Therefore, these compounds constitute a danger to human health when consumed in excess. The quantity of nitrates and nitrites in root vegetables is dependent on the soil and environment, including water and fertilizers. Danger related to the consumption of nitrates and nitrites appears when high doses are taken in. It is necessary to raise awareness of nitrates and nitrites among consumers who eagerly purchase dietary supplements with beetroot or other preparations containing these compounds. 

### 5.2. Heavy Metals

Among several factors that affect the migration of contaminants in soil are the pH, ionic strength, soil texture, organic matter content, and time. Soil quality is a key factor in reducing the pollution of root vegetables. The most common toxic metals are Pb and Cd [160]. The Joint FAO/WHO Expert Committee on Food Additives (JECFA) established a provisional tolerable monthly intake (PTMI) for Cd (25 μg/kg b.w.) [161]. However, for Pb, it was concluded that it was not possible to establish a provisional tolerable weekly intake (PTWI) that would be considered safe for human health [162]. Despite the lack of designation of a safe dose of Pb, the European Commission has set permissible amounts of this element in vegetables. The concentration of Pb in root vegetables cannot be higher than 0.3 mg in 100 grams of fresh weight, while that of Cd cannot exceed 0.2 mg/100 g [163]. The average adult person in Europe consumes from 0.36 up to 2.43 µg/kg b.w./day of Pb through dietary sources [164]. 

The contamination of vegetables is mainly determined by the pollution of the environment. Soil along the Msimbazi River valley in the city of Dar es Salaam (Tanzania) was examined and the levels of chromium (Cr)(VI), Pb, copper (Cu), and Cd were determined. The highest concentrations were found for Cr (1.14 mg/L) and Pb (1.113 mg/L). The concentration of Pb was higher than the limits for rivers set at 0.01mg/L by the Food and Agricultural Organization and the World Health Organization (FAO/WHO). The study showed that the metal content was higher in the surface layers of the soil [165]. 

In addition, soil quality depends on many factors, with the most important being the location and irrigation method. Selected vegetables harvested from different geographic areas were examined. The same plants grown in different soils were characterized by various Cd levels. Its level in turnips grown in soil irrigated with wastewater was 0.78 mg/kg, while that in turnips grown near the Dabaoshan mine in Guangdong, China, amounted to 2.9 mg/kg, and that in turnips grown near the same mine, but in Zhongxin City, was 0.28 mg/kg [166]. 

The penetration of vegetables by contaminants from the soil is mainly related to the phenomenon of absorption. It has been shown that this process for some heavy metals, such as Cr(VI), is proportional to their concentration in the soil, while desorption is inversely proportional. This means that, as the concentration of Cr in the soil increases, the plant removes this toxic metal from its body more and more slowly. In addition, soil pH is important for the absorption of each element. The highest percentage of absorption for Cr(VI) was achieved at pH 3, and that for Pb was achieved at pH 5. This is related to the chemical form in which the metals occur (salts) and their solubility at the corresponding pH for ions [148]. The presence of soluble heavy metals, such as Pb and Cr(VI), in coal ash is one of the main reasons for groundwater pollution and subsoil degradation. A difference in the accumulation of heavy metals (e.g., zinc) in different parts and species of plants has been observed. A higher content of heavy metals was noted in the skin of the roots than in the flesh of plants [167]. 

The toxicity of these metals includes the production of reactive oxygen species, reduction in antioxidant defenses, inactivation of enzymes, and increased oxidative stress [168]. Moreover, together with higher urbanization, vegetables are more exposed to heavy metals due to environmental pollution. Therefore, there is a need for the constant evaluation of vegetable contamination and human risk assessment. 

### 5.3. Pesticides

Residues of plant-protection products can be found in vegetables, including root vegetables. Their content in vegetables should not exceed the maximum residue levels (MRLs). However, incidental cases of MRLs being exceeded in root vegetables were found in random crops [12,169]. However, the application of pesticides, as recommended in field tests, resulted in no exceedance of the MRLs [12]. Root vegetables appear to be safe in terms of pesticide residues. However, in a Pesticide Action Network Europe (PANEurope) report for 2011–2019 published in May 2022, the top ten vegetables most frequently found to have pesticide residues were celery, parsley, and parsnips. Root vegetables require at least washing, and often peeling, blanching, boiling, or frying, before they can be eaten or processed. These processes, as a meta-analysis by Keikotlhaile et al. (2010) showed, reduce pesticide residues in vegetables, making them safer to eat [170]. 

### 5.4. Polycyclic Aromatic Hydrocarbons

PAHs constitute another example of organic compounds that contaminate root vegetables. The most dangerous PAHs include benzopyrene (BaP), which, like other PAHs, can be absorbed by plant roots [171]. The compounds’ accumulation is not the same for all root vegetables. Ashraf and Salam (2012) found a lower total PAH content in turnips (9.26 µg/kg f.w.) compared with carrots (11 µg/kg f.w.) [14]. Moreover, the scientists noted that more PAHs were accumulated in the peel than the core [14].

### 5.5. Microplastics

Microplastics (MPs) are a pollutant of global concern [172,173]. They are defined as synthetic materials with a high polymer content [174]. Plastic microparticles have a size of 0.1 to 5000 µm. Particles smaller than MPs with a size between 1 and 100 nm are called nanoplastics (NPs) [175].

Plastic particles can enter terrestrial ecosystems and accumulate in the soil [176]. They affect the enzymatic activity of the soil and the microorganisms present within it [177]. Plastic pollution of cultivated soils is mainly related to agricultural activities, such as the use of plastic tunnels, compost fertilization, and the use of sewage sludge [172,178]. The MP contents in soils from different regions of the world, such as Spain, Brazil, and China, were analyzed and found to be 2000 [178], 10,782 [179], and 52,081.7 particles/kg [180]. It has been found that polyethylene (PE) and polypropylene (PP) particles are the most commonly found MPs in soil [179,181]. These contaminants are usually in the form of fibers and fragments [176,181,182]. Microplastics can migrate from the soil into plants. They are capable of penetrating seeds, roots, stems, leaves, and fruits, depending on the type and size of the particles, and the plant species [182,183]. The uptake of plastic particles is inversely proportional to their size [184]. 

Microplastics can be a contaminant of edible vegetables, including the common carrot *Daucus carota*. Plastic particles, ranging in size from 1.36 to 2.00 µm, have been found in this vegetable in amounts of 72,175 to 130,500 particles/g [185,186]. The MP particles are absorbed into the carrot root from the environment. The area of absorption is increased by microscopic hairs located on the outer side of the epidermis of the central root of *Daucus carota* [185]. Polystyrene (PS) particles of 1 μm can accumulate in the intercellular layer after entering the carrot root, but are unable to penetrate cells. However, particles of 0.2 μm can migrate into the leaves. They destroy the tertiary structure of pectin methylesterase in carrots, which is involved in the synthesis of cell wall components, contributing to the loss of the vegetable’s crunchiness. Microplastics reduce the nutritional value of *Daucus carota* and pose potential risks to human health [187,188]. 

The common radish *Raphanus sativus* can accumulate, in its roots, exogenously supplied MP. It was found that poly(acrylonitrile-co-butadiene-co-styrene) (ABS) particles, up to 2 µm in size, could penetrate the intercellular spaces of root cells [185]. Similarly to PS, it can enter the intercellular spaces of carrots [185]. In addition, ABS, through the small fissures from which radish lateral roots emerge, can penetrate the xylem vessels [188]. It has also been found that PS particles with a size of 100 nm affect the reduction in the root length of *Raphanus sativus* [188].

Microplastics are a new dangerous contaminant of food. There is a need to create unified methods of their determination in soil and root vegetables. Studies are focused on the migration of MPs to root vegetables from intentionally contaminated soil. There is a lack of research evaluating the content of MPs in traditional cultivation. Moreover, the topic of MP migration into root vegetables during storage, freezing, and high-temperature exposure during contact with various types of artificial packaging should be expanded.

## 6. Conclusions

This article outlines the risks and benefits of root vegetable consumption and characterizes the chemical compositions of selected plants in this group. Root vegetables are widely available all year and relatively inexpensive for the average consumer. Several of them constitute popular sources of health-promoting substances. Carrots are known as a source of β-carotene, beetroots constitute a source of betalains, and parsley is understood to be a source of Ca. These vegetables are characterized by high bioactivity. All of the described root vegetables share common characteristics, such as high antioxidant potential due to their high contents of phenols and flavonoids. These compounds exhibit health-promoting effects, but excess levels can result in negative outcomes. The comparison of antioxidant effects is difficult due to the lack of standardization among methods used for their analysis. Therefore, there is a need for a reference method that would allow for correct interpretation. Moreover, studies in humans show that the consumption of root vegetables has many health properties. The best-examined is carrot and its influence on cancer prevention, and parsley’s preventive effect on kidney stones. There are plenty of human studies concerning beetroot and its impact on respiratory infections or vascular functioning. However, the results are contradictory; thus, further research on larger groups is required to draw reliable conclusions.

The bioavailability of antioxidant compounds can be high due to fermentation in the colon. This is another aspect that requires further research. The effects of traditional vegetable processing techniques, such as cooking, are quite well known. However, the influence of fermentation on this group of vegetables is poorly understood. Nowadays, more and more of these products are being used for silage production, so this topic should be further explored in the future. 

Roots can be contaminated by various substances (heavy metals, PAHs, nitrates and nitrites, or microplastics) due to their morphological structure and growth in soil. However, their levels are not as high as that required to be dangerous to human health. Moreover, culinary processing, such as peeling, or cooking, reduce the contents of contaminants by more than 50%. Currently, the emerging problem is microplastics. They are a new danger to human health and still little is known about their effects. Therefore, there is a need for a standardized method for the detection of microplastics in vegetables and evaluations of their toxicity to humans. There is a need to establish permissible standards for their content in food, including root vegetables.

## Figures and Tables

**Table 1:** Composition of root vegetables [5,6].

Components	Carrot	Beetroot	Celery, Root	Parsley, Root	Radish	Turnip	Horseradish
Aqua (g/100 g)	88.3	87.6	88.0	85.3	95.3	91.9	75.0
Energy (kcal/100 g)	41.0	43.0	42.0	49.0	16.0	28.0	81.0
Proteins (g/100 g)	0.93	1.61	1.5	2.6	0.68	0.9	4.5
Lipids (g/100 g)	0.24	0.17	0.3	0.5	0.1	0.1	0.6
Carbohydrates (g/100 g)	9.58	9.56	9.2	10.5	3.4	6.43	18.1
Dietary fiber, total (g/100 g)	2.8	2.8	1.8	4.2	1.6	1.8	7.3
Calcium (mg/100)	33.0	16.0	43.0	43.0	25.0	30.0	78.0
Iron (mg/100 g)	0.3	0.8	0.7	1.1	0.34	0.3	1.2
Magnesium (mg/100 g)	12.0	23.0	20.0	27.0	10.0	11.0	43.0
Phosphorus (mg/100 g)	35.0	40.0	115.0	77.0	20.0	27.0	120.0
Potassium (mg/100 g)	320.0	325.0	300.0	339.0	233.0	191.0	740.0
Sodium (mg/100 g)	87.0	78.0	100.0	49.0	39.0	67.0	7.0
Zinc (mg/100 g)	0.24	0.35	0.33	0.6	0.28	0.27	1.40
Copper (mg/100 g)	0.06	0.075	0.07	0.14	0.05	0.085	0.23
Selenium (µg/100 g)	0.1	0.7	0.7	n.d.	0.6	0.7	n.d.
Vitamin C (mg/100 g)	5.9	4.9	8	45.0	14.8	21.0	114.0
Thiamin (mg/100 g)	0.066	0.031	0.05	0.1	0.012	0.04	0.14
Riboflavin (mg/100 g)	0.058	0.4	0.06	0.086	0.039	0.03	0.11
Niacin (mg/100 g)	0.983	0.334	0.7	2.0	0.254	0.4	0.6
Pyridoxine (mg/100 g)	0.138	0.067	0.165	0.23	0.071	0.09	0.18
Folate, total (µg/100 g)	19.0	109.0	8.0	180.0	25.0	15.0	37.0

n.d.—no data.

**Table 2:** Botanical families of root vegetables [21].

Botanical Family	*Apiaceae*	*Amaranthaceae*	*Brassicaceae*
Vegetable name	Carrot Parsley Celery	Beetroot	Radish Turnip Horseradish

**Table 5:** Influence of root vegetables from the botanical order *Amaranthaceae* on health.

Vegetable	Type of Study	Form of Vegetable/Compound Tested	Effect	Source
Beetroot	In vivo/people/ meta-analysis	Beetroot juice	General systolic blood pressure was lower among people with beetroot juice supplementation Potential nitrate-independent effect of beetroot juice	[127]
Beetroot	In vivo/people/ meta-analysis	Various beetroot products	Beetroot supplementation offered no significant improvement to peak or mean power output during HIIT (high-intensity interval training) or SIT (sprint interval training)	[128]
Beetroot	In vivo/people/ meta-analysis	Beetroot	Beetroot consumption was associated with an improvement in vascular function Effect on endothelial function was significantly associated with the dose of inorganic nitrates	[129]
Beetroot	In vivo/people	Beetroot juice	Ingestion of beetroot juice increased blood flow to the brain and enhanced exercise performance Older adults who exercised and consumed beetroot juice demonstrated greater consistency within the motor community and fewer secondary connections with the insular cortex compared with those who exercised without beetroot juice	[130]

**Table 6:** Influence of root vegetables from the botanical order *Brassicaceae* on health.

Vegetable	Type of Study	Form of Vegetable/Compound Tested	Effect	Source
Radish	In vivo/mice In vitro/PC_12_ cells	Radish extract	Increased spontaneous alternation behaviors and step-through latency Reduced lipid peroxidation and A*β* aggregation in a biochemical study of mice brain tissues Attenuated H_2_O_2_-induced oxidative stress in cells	[143]
Radish	In vivo/people	Radish, root	Increased calcium oxalate excretion in both women and men	[144]
Radish	In vivo/men	Diet supplement with black radish extract	Increased activity of phase I and phase II liver enzymes after 4 weeks of supplementation	[145]
Turnip	In vivo/people	*Brassica rapa* L.–turnip powder	Peak O_2_ pulse and peak VO_2_/kg significantly improved after 7-day turnip consumption during the Bruce treadmill test Antioxidant activity improved after 7 days of intervention	[137]
Horseradish	In vitro/human lymphocytes	Horseradish root extract with kaempferol or quercetin added	Both extracts with kaempferol and quercetin decreased DNA damage caused by H_2_O_2_	[146]
Horseradish	In vivo/people	Horseradish, root	No thermogenesis effect noted Heart rate decreased Diastolic blood pressure increased No effect on appetite noted	[147]
Horseradish	In vitro/ human peripheral blood mononuclear cells (PBMC)	Horseradish extract	Extract concentration-dependent inhibition of anti-inflammatory response to lipopolysaccharide (LPS) in terms of TNF-*α* release	[141]
